# Metabolic Effects of Ketogenic Diets and Their Utilization in Obesity Management: A Systematic Review

**DOI:** 10.7759/cureus.36720

**Published:** 2023-03-26

**Authors:** Vani Malhotra, Anupama Sawal

**Affiliations:** 1 Anatomy, Jawaharlal Nehru Medical College, Datta Meghe Institute of Medical Sciences, Wardha, IND

**Keywords:** metabolism, obesity, ketosis, carbohydrates, fats, ketogenic diet

## Abstract

A ketogenic diet (KD), more commonly called a "keto" diet, is a dietary regime that focuses on reducing carbohydrates and replacing them with healthy fats. It has proven to improve health and has resurfaced as a trendy weight loss method. Keto, in simple terms, works by mimicking starvation and forcing the body to utilize and deplete fat as its core energy source instead of its usual source of glucose (sugar). More technically, it gives ignition to a process called ‘ketosis’. Ketosis is the process of generation of ketone bodies when the liver metabolizes fat. There are several versions of this diet, each of which addresses slightly variable issues as well as hones unique requirements. Individuals will require a unique combination of fat, carbohydrates, and protein depending on their genetic and physical makeup. The advantages and hazards of using the KD to manage obesity are examined in this review of the literature.

## Introduction and background

**Figure 1:**
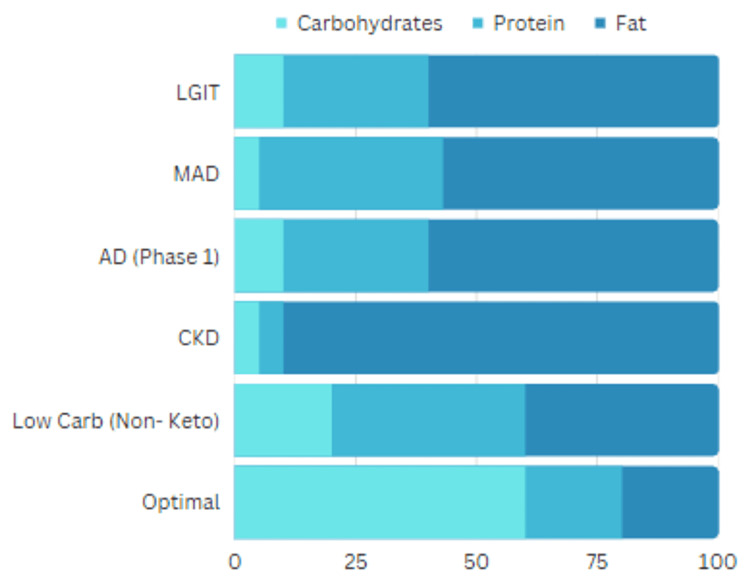
Distribution of nutrients in different types of KD The figure was created by the author and hence not subject to copyright AD: Atkins Diet; CKD: Classical Ketogenic Diet; KD: ketogenic diet; LGIT: Low Glycemic Index Treatment; MAD: Modified Atkins Diet

The ketogenic diet (KD) is a systemic diet in which foods rich in fats and moderate protein are consumed and foods rich in carbohydrates are avoided. The technique was first developed at the Mayo Clinic by Dr. Russel Wilder and has been used for many decades to treat epilepsy in children (with regard to refractory seizures) [1,2]. The lack of carbohydrates in food intake results in the oxidation of fatty acids known as ketogenesis. Ketogenesis is a biochemical pathway that yields ketone bodies [3], the body's alternate source of energy. KD imitates the metabolic consequences of fasting in this way. The proportion of fats, carbohydrates, and proteins needed in a diet to induce ketosis varies from individual to individual depending on genetics, environmental factors, activity levels, etc. but is calculated on the basis of the type of KD followed. Classical KD (CKD), Medium-Chain Triglyceride KD, Atkins Diet (AD), Modified Atkins Diet (MAD), and Low Glycemic Index Treatment (LGIT) [4] can all be categorized based on the various aspects (Figure 1). Ketogenesis is regulated more favorably by glucagon than by insulin, which can inhibit ketone production [5]; glucagon and insulin are hormones secreted by alpha and beta cells of the pancreas, respectively, and are regulators of blood glucose levels. Glucagon functions to increase glucose levels in the blood in times of need, whereas insulin acts antagonistically.

Obesity is an epidemic that is rapidly growing worldwide [6] and has many implications. Obesity, a disorder involving excessive body fat, puts people at a higher risk of elevated blood pressure and cholesterol levels and hence results in comorbidities such as cardiovascular diseases, arthritis, ovarian disorders (PCOS), sleep apnea, and diabetes mellitus to name a few. It also results in the aggravation of conditions such as asthma. Furthermore, it also leads to difficulties in performing various day-to-day activities and lowers life expectancy and quality of life. A key step promoted in the prevention of chronic illnesses that are linked to high morbidity and mortality in industrialized nations is weight loss [7]. Even though weight management is often encouraged by healthcare organizations, this objective frequently fails. Excessive weight gain is caused by a combination of genetic susceptibility, sedentary lifestyles, and high-calorie intake. Although the premise that dietary changes and adjustments in activity levels are necessary to assist weight reduction and weight control may be widely accepted, there is still disagreement regarding the appropriate amount and kind of exercise as well as the ideal diet [8,9]. The exercise prescription should be individualized taking into account several factors including comorbidities that can be contraindications to certain exercise forms. A general consensus has been reached that a disparity between energy intake and expenditure as per daily requirements is what causes obesity, and a variety of genetic, socioeconomic, and cultural variables all contribute to obesity [10], which is a complex disease. Numerous genes have been linked to adiposity and weight growth, demonstrating the high heritability of obesity [11,12]. Reduced physical activity, insomnia, hormonal issues, pharmaceutical and recreational drug usage, increasingly detrimental eating habits, and intake of large amounts of refined foods with high sugar values and hindered energy metabolism are some other reasons for obesity [9]. This is where the role of the high-fat and low-calorie KD comes into play.

## Review

Search methodology

**Figure 2:**
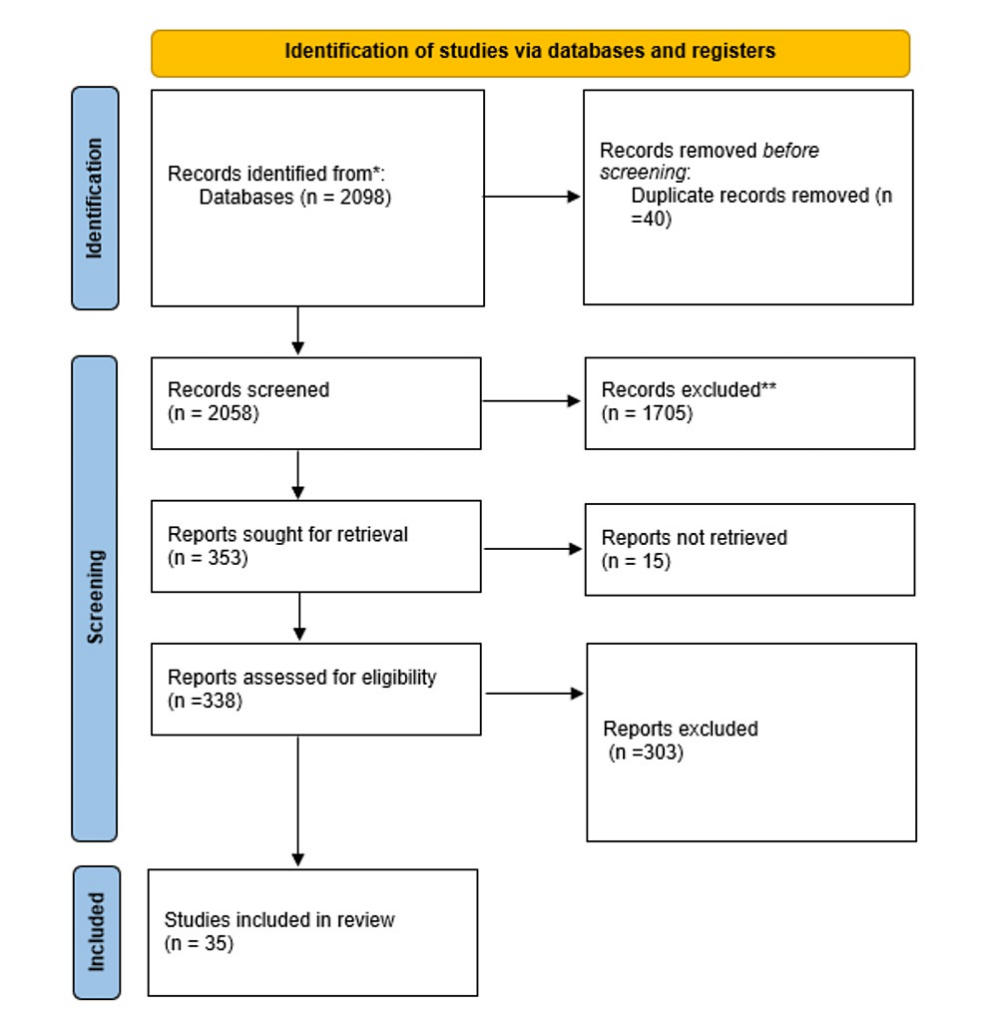
PRISMA flow diagram depicting the selection of studies PRISMA: Preferred Reporting Items for Systematic Reviews and Meta-Analyses

We undertook a systematic search through PubMed Central in September 2022 using keywords such as "metabolism" and "ketogenic diet" (((metabolism [Title/Abstract]) OR ("metabolism" [MeSH Terms]) AND (("ketogenic diet" [Title/Abstract]) OR (KD [Title/Abstract])) OR ("ketogenic diet" [MeSH Terms]). We additionally searched for key references from bibliographies of the relevant studies. The search was updated in January 2023. One reviewer independently evaluated the retrieved studies against the inclusion criteria, in the beginning, based on the title and abstract and then on full texts. Another reviewer also reviewed approximately 20% of these studies to validate the inclusion of respective studies. Preferred Reporting Items for Systematic Reviews and Meta-Analyses (PRISMA) guidelines were followed for this study (Figure 2).

Physiological principles of ketogenic diets

Ketones are chemical compounds distinguished typically by the occurrence of a carbonyl group and have the chemical formula RC (=O) R′, where two R groups of the structure can be any carbon-containing substituents. Although mitochondria of hepatocytes of the liver are the primary producers of ketones in the human body [13], most body tissues can utilize ketones as an alternative source of energy. The primary ketones formed by hepatocytes are acetoacetate followed by acetone, and 3-hydroxybutyrate although technically 3-hydroxybutyrate is a hydroxy fatty acid. As described by Krebs, KD induces a state of "physiological ketosis" which is especially different from the pathological state of ketosis in diabetic conditions [14]. Usually, the sole fuel for the functioning of the brain is glucose [15], and fatty acids cannot be used as an energy source, as they are unable to cross the blood-brain barrier [16].

**Figure 3:**
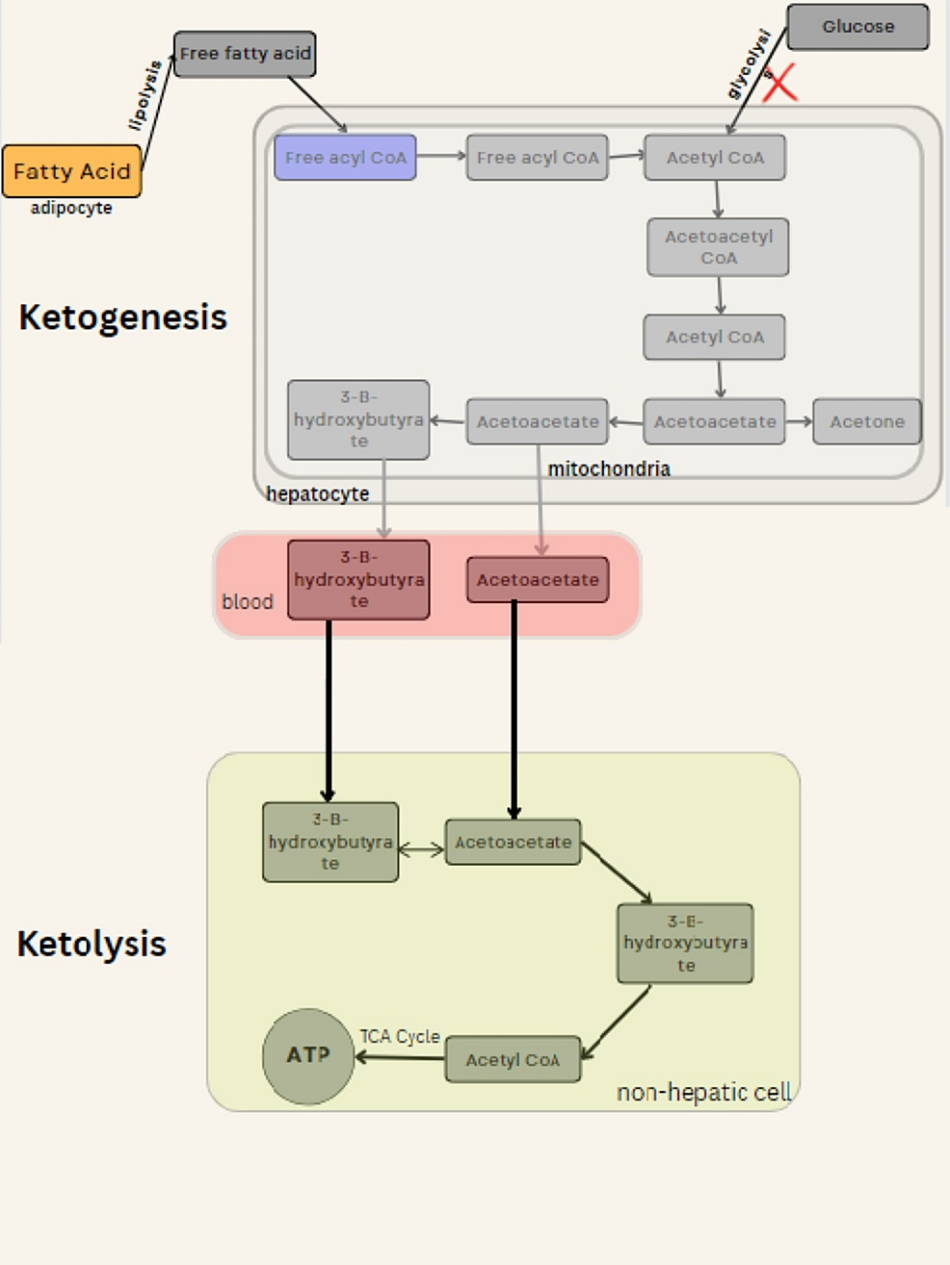
Mechanism of the ketogenic diet This image was created by the author and hence not subject to copyright

The body's glucose stores run out after a few days of fasting or a dramatically decreased carbohydrate diet (less than 20 grams a day), making it difficult to produce oxaloacetate for regular Krebs cycle fat oxidation and to provide glucose to the brain (central nervous system) [17,18]. The process of production and breakdown of ketones known as ketogenesis and ketolysis respectively are regulated by the release of hormones insulin and glucagon. Ketogenesis is regulated more favorably by glucagon than by insulin, which can inhibit ketone production [5]. Fatty acids are first converted into free fatty acids through lipolysis, which is followed by their transfer to hepatocyte (liver cell) from adipocyte (fat cell) and oxidation to acetyl coenzyme A (CoA). Acetoacetyl CoA is produced when the enzyme thiolase converts acetyl CoA into acetoacetyl CoA in low-glucose settings. Next, the enzyme HMG CoA synthase hastens the conversion of acetoacetyl CoA to hydroxymethylglutaryl CoA (HMG CoA). HMG CoA is then changed into acetoacetyl CoA and acetoacetate by the enzyme HMG CoA lyase. With the help of 3-hydroxybutyrate hydrogenase, acetoacetate can be further transformed into acetone or 3-hydroxybutyrate. Acetone is no longer used and is thus eliminated primarily through the lungs and partly through urine, whereas 3-hydroxybutyrate and acetoacetate can both diffuse into the bloodstream at the same time and reach non-hepatic organs. To actually enable the use of ketone bodies as the primary form of energy, ketolysis is necessary. Succinyl CoA: 3-oxoacid CoA transferase (SCOT) and acetyl CoA acetyltransferase are enzymes that are used to convert acetoacetate and 3-hydroxybutyrate back to acetyl CoA during the ketolysis process (ACAT1). After completing the Krebs cycle, acetyl CoA is further oxidized, producing 22 molecules of ATP. Even though the liver is the primary generator of ketones, the inability to utilize them due to the lack of SCOT makes ketolysis more prevalent in non-hepatic cells (Figure 3) [19,20].

Stages of the ketogenic diet

The protocol of the KD is divided into three stages according to a study conducted by Muscogiuri et al. titled "The management of very low-calorie ketogenic diet in obesity outpatient clinic: a practical guide" [21]- 

Active Stage

The consumption of 600-800 kcal/day of calories a day, fewer carbohydrates, and a higher amount of lipids are the defining characteristics of the active period (usage of healthier oils). High-biological-value proteins are required for every kilogram of appropriate body weight in order to retain proper bodily functioning. Patients in this stage consume high-biological-value protein dishes like boiled chicken, chickpeas, lentils, and dairy products five times daily together with low-glycemic produce during phase 1 of this stage, which is then divided into three ketogenic phases. One of the protein portions in phase 2 at either lunch or supper is exchanged with a leaner protein [22]. The biological protein preparation is switched for a second portion of protein low in fat in the third phase. According to international recommendations, patients should include micronutrients and minerals adequately in their diet as well [23]. This stage of activity and change is maintained until the patient has successfully dropped approximately 80% of the desired amount of weight to be lost. Because of this, the length of the ketogenic phases varies based on the person and the weight loss goal. Typically, the active period lasts for a total of 8-12 weeks.

Re-education Stage

After the introductory phases, the patient is switched to a low-calorie diet during this second stage. Here onwards, the patients will gradually reintroduce dietary diversity while engaging in an alimentary re-education program to help them maintain their weight over the long term. Fruit and dairy products (phase 4) are the first to be progressively re-established, followed by legumes (phase 5) and bread, pasta, and cereals (phase 6), in increasing order of their glycemic values [22,24]. Progressing toward the latter steps, the daily caloric goal value lies between 800-1500 kcal.

Maintenance Stage

Following the aforementioned stages of food reintroduction, there is a maintenance phase that involves following a diet that is balanced in terms of carbs, protein, and fat. This phase's key goals are to maintain weight loss and encourage an overall healthy lifestyle [25].

Limitations and side effects

Most research regarding KD for managing obesity that used very-low-calorie ketogenic diets (VLCKDs) have daily calorie limits of 500-800. It can be proposed that the changes noted are due to the relatively low calories and not the actual nutrition and nature of the food consumed, specifically because a majority of the studies did not include control groups to accurately evaluate the objective effect of the different nutritional composition of a diet as shown by repeated studies [26,27,28]. Restrictive diets should always be administered with proper professional guidance; otherwise, the associated chances of failure and risk of side effects are considerably more [29]. The American Heart Association and the Dietary Guidelines for Americans advise against emphasizing foods rich in saturated/unhealthy fat, and it may have a detrimental effect on blood LDL cholesterol. One could modify the diet by emphasizing foods with healthy fats and lacking saturated fat, like olive oil, nuts, seeds, avocados, and fish. To avoid nutritional deficiencies, one can construct a meal plan that is personalized to one's present health conditions, and carefully note any biochemical effects after initiating the diet; it is advisable that anybody who intends to start KD speaks with their doctor and a nutritionist. 

When it comes to resuming carbohydrates after weight reduction, advice should be sought from a dietician; however, some general recommendations are to include a variety of meats, fruits and vegetables, berries, and roughage in your diet on a daily basis to ensure that you are getting the micronutrients (minerals, and vitamins) that are typically easily available in grains like wheat but have been cut out of your diet to prevent side effects including headache, foggy brain, fatigue, irritability, nausea, difficulty sleeping, and constipation, commonly known as "the keto flu". Safe nutritional ketosis levels are 1.5-3.0 mmol/l as recommended by researchers Stephen Phinney and Jeff Volek. Ketone levels can be measured through urine and blood tests. Nutritional intervention cases, reports, and studies are generally difficult to carry out in humans due to the issue of a high level of participant variability and this is a predicament with which many researchers struggle. Gender plays a major role in the outcomes of treatment plans as shown by studies [30,31]. Additionally, it might be challenging to maintain hormonal balance, especially in females [32]. The results of a dietary study may be influenced by thyroid diseases, pregnancy, and lactation, which are frequent issues in studies conducted among women. Not only gender-linked hormonal and cyclical issues, but also the body type (avocado, pear, apple, etc.) may predispose to varying results [33]. The mean effects of the intervention may vary depending on the disease class and original body weight in the research on obesity. BMI is thought to be the principal variable in many studies on obesity. However, more and more research is showing that BMI may not be the most accurate standard to account for the characteristics of obesity [34]. It is argued that body composition, rather than body weight, should be used to classify obesity [35]. Some contraindications are seen in patients with diabetes who inject insulin or take oral hypoglycemic medications (there is a risk of developing serious hypoglycemia if their medication is not properly dosed prior to starting a diet due to already low blood sugar levels) as well as medications such as SGLT2 inhibitors.

## Conclusions

For obese patients who have tried ineffective diets to reduce unhealthy weight rapidly, KD is often a useful therapeutic strategy. Investigations into metabolism and assessments of hormonal changes, etc. are all crucial. Improved controls for diet adherence should be used in more randomized, carefully conducted research on metabolism, related effects, and control of dietary composition. This diet may provide hope for people who have had trouble losing weight in other ways. The removal of many foods and the likelihood of adverse symptoms may also make sticking to a disciplinary diet regime difficult. To avoid nutritional deficiencies, one should construct a meal plan that is personalized to one's present health conditions, and carefully note any biochemical effects after initiating the diet; it is advisable that anybody who intends to start KD speaks with their doctor and a nutritionist. When it comes to resuming carbohydrates after weight reduction, advice should be sought from a dietician. In addition to emphasizing healthy high-fat foods, it is important to include a variety of meats, fruits and vegetables, berries, and roughage in your diet on a daily basis to ensure that you're getting the micronutrients (minerals, and vitamins) that are typically easily available in grains like wheat but have been cut out of your diet. Secondly, clinical signs such as nausea, exhaustion, irritability, fatigue, indigestion, vomiting, and constipation should be addressed with a medical evaluation by professionals as well as nutritionists, Lastly, people with diabetes who inject insulin or take oral hypoglycemic medications must consider that they run the risk of developing serious hypoglycemia if their medication is not properly dosed prior to starting a diet. This is due to low blood sugar levels caused by following KD.

## References

[1] Kim JM (2017). Ketogenic diet: old treatment, new beginning. Clin Neurophysiol Pract.

[2] Sampaio LP (2016). Ketogenic diet for epilepsy treatment. Arq Neuropsiquiatr.

[3] Dhillon KK, Gupta S (2023). Biochemistry, Ketogenesis. https://pubmed.ncbi.nlm.nih.gov/29630231/.

[4] Barzegar M, Afghan M, Tarmahi V, Behtari M, Rahimi Khamaneh S, Raeisi S (2021). Ketogenic diet: overview, types, and possible anti-seizure mechanisms. Nutr Neurosci.

[5] Drabińska N, Wiczkowski W, Piskuła MK (2021). Recent advances in the application of a ketogenic diet for obesity management. Trends Food Sci Technol.

[6] Ahirwar R, Mondal PR (2019). Prevalence of obesity in India: a systematic review. Diabetes Metab Syndr.

[7] Cohen JB (2017). Hypertension in obesity and the impact of weight loss. Curr Cardiol Rep.

[8] Fanti M, Mishra A, Longo VD, Brandhorst S (2021). Time-restricted eating, intermittent fasting, and fasting-mimicking diets in weight loss. Curr Obes Rep.

[9] Panuganti KK, Nguyen M, Kshirsagar RK (2022). Obesity. StatPearls - NCBI Bookshelf.

[10] Jalali MS, Sharafi-Avarzaman Z, Rahmandad H, Ammerman AS (2016). Social influence in childhood obesity interventions: a systematic review. Obes Rev.

[11] Campbell LV (2017). Genetics of obesity. Aust Fam Physician.

[12] Thaker VV (2017). Genetic and epigenetic causes of obesity. Adolesc Med State Art Rev.

[13] Chavan R, Feillet C, Costa SS, Delorme JE, Okabe T, Ripperger JA, Albrecht U (2016). Liver-derived ketone bodies are necessary for food anticipation. Nat Commun.

[14] Krebs HA (1966). The regulation of the release of ketone bodies by the liver. Adv Enzyme Regul.

[15] Hartman AL, Gasior M, Vining EP, Rogawski MA (2007). The neuropharmacology of the ketogenic diet. Pediatr Neurol.

[16] Daneman R, Prat A (2015). The blood-brain barrier. Cold Spring Harb Perspect Biol.

[17] Owen OE, Morgan AP, Kemp HG, Sullivan JM, Herrera MG, Cahill GF Jr (1967). Brain metabolism during fasting. J Clin Invest.

[18] Owen OE (2005). Ketone bodies as a fuel for the brain during starvation. Biochem Mol Biol Educ.

[19] Fukao T, Mitchell G, Sass JO, Hori T, Orii K, Aoyama Y (2014). Ketone body metabolism and its defects. J Inherit Metab Dis.

[20] Kolb H, Kempf K, Röhling M, Lenzen-Schulte M, Schloot NC, Martin S (2021). Ketone bodies: from enemy to friend and guardian angel. BMC Med.

[21] Muscogiuri G, Barrea L, Laudisio D, Pugliese G, Salzano C, Savastano S, Colao A (2019). The management of very low-calorie ketogenic diet in obesity outpatient clinic: a practical guide. J Transl Med.

[22] Larsen TM, Dalskov SM, van Baak M (2010). Diets with high or low protein content and glycemic index for weight-loss maintenance. N Engl J Med.

[23] Prudencio MB, de Lima PA, Murakami DK, Sampaio LP, Damasceno NR (2021). Micronutrient supplementation needs more attention in patients with refractory epilepsy under ketogenic diet treatment. Nutrition.

[24] Atkinson FS, Brand-Miller JC, Foster-Powell K, Buyken AE, Goletzke J (2021). International tables of glycemic index and glycemic load values 2021: a systematic review. Am J Clin Nutr.

[25] Frank-Podlech S, Watson P, Verhoeven AA, Stegmaier S, Preissl H, de Wit S (2021). Competing influences on healthy food choices: mindsetting versus contextual food cues. Appetite.

[26] Castro AI, Gomez-Arbelaez D, Crujeiras AB (2018). Effect of a very low-calorie ketogenic diet on food and alcohol cravings, physical and sexual activity, sleep disturbances, and quality of life in obese patients. Nutrients.

[27] Hall KD, Chen KY, Guo J (2016). Energy expenditure and body composition changes after an isocaloric ketogenic diet in overweight and obese men. Am J Clin Nutr.

[28] Schiavo L, Pilone V, Rossetti G, Barbarisi A, Cesaretti M, Iannelli A (2018). A 4-week preoperative ketogenic micronutrient-enriched diet is effective in reducing body weight, left hepatic lobe volume, and micronutrient deficiencies in patients undergoing bariatric surgery: a prospective pilot study. Obes Surg.

[29] Caprio M, Infante M, Moriconi E (2019). Very-low-calorie ketogenic diet (VLCKD) in the management of metabolic diseases: systematic review and consensus statement from the Italian Society of Endocrinology (SIE). J Endocrinol Invest.

[30] Mohorko N, Černelič-Bizjak M, Poklar-Vatovec T, Grom G, Kenig S, Petelin A, Jenko-Pražnikar Z (2019). Weight loss, improved physical performance, cognitive function, eating behavior, and metabolic profile in a 12-week ketogenic diet in obese adults. Nutr Res.

[31] Comitato R, Saba A, Turrini A, Arganini C, Virgili F (2015). Sex hormones and macronutrient metabolism. Crit Rev Food Sci Nutr.

[32] Kapoor E, Faubion S, Hines S, Stuenkel CA (2017). Women's health endocrine update. J Womens Health (Larchmt).

[33] Christakoudi S, Tsilidis KK, Evangelou E, Riboli E (2021). Association of body-shape phenotypes with imaging measures of body composition in the UK Biobank cohort: relevance to colon cancer risk. BMC Cancer.

[34] Mahadevan S, Ali I (2016). Is body mass index a good indicator of obesity?. Int J Diabetes Dev Ctries.

[35] De Lorenzo A, Soldati L, Sarlo F, Calvani M, Di Lorenzo N, Di Renzo L (2016). New obesity classification criteria as a tool for bariatric surgery indication. World J Gastroenterol.

